# Instability-Induced Pattern Transformation in Soft Metamaterial with Hexagonal Networks for Tunable Wave Propagation

**DOI:** 10.1038/s41598-018-30381-1

**Published:** 2018-08-07

**Authors:** Chao Gao, Viacheslav Slesarenko, Mary C. Boyce, Stephan Rudykh, Yaning Li

**Affiliations:** 1 Department of Mechanical Engineering, University of New Hampshire, Durham, NH USA; 2 Faculty of Aerospace Engineering, Technion–Israel Institute of Technology, Haifa, Israel; 3 School of Engineering and Applied Science, Columbia University, New York, 10027 USA; 4 Department of Mechanical Engineering, University of Wisconsin-Madison, Madison, WI USA

## Abstract

Instability-induced pattern transformations of the architectured multi-phase soft metamaterial under bi-axial compression were explored. The soft metamaterial is composed of two phases: a soft matrix and a reinforcing hexagonal network embedded in the matrix. Equi-biaxial loading is found to induce both micro- and macro- instabilities in the networked architecture. Two types of instability patterns were observed, dependent upon the architecture geometry and the material combination. The critical strain for triggering instability and the two resulting types of patterns was derived, and a theoretical criterion for the transition between the two patterns was determined. Type I patterns retain the original periodicity of the architecture but wrinkles the network walls whereas Type II patterns transform the overall periodicity of the architecture while bending the network walls. Elastic wave propagation analysis was performed for the two distinct patterns under both stressed and stress-free conditions: a change in band gaps is found for both instability-induced pattern transformations, but differs for each type due to their dramatic difference in structure transformation (i.e. Type I wall wrinkling vs. Type II periodicity switching). The distinguished mechanical behavior and the rich properties of this category of multi-phase soft metamaterial can be used to design new smart materials with switchable functionalities controllable by deformation.

## Introduction

Under larger elastic deformation, architectured soft metamaterials can experience dramatic pattern transformation triggered by structural and material instability. Recognizing that patterned materials can exhibit tailored wave propagation behaviour^1–3^, the instability-induced reversible pattern transformation has been to provide deformation-dependent switchable mechanical and wave propagation properties^4–6^. By tuning the geometry and material combination of the soft metamaterials, both the critical strain and the corresponding pattern transformation can be tailored, and thus the wave propagation properties can be tuned accordingly. Therefore, through geometrical design and the selection of the constituent materials, these soft metamaterials can be used to design tunable mechano-adaptive composites. These soft architectured composites will provide multifunctional capabilities to allow for tunable properties, such as thermal and wave propagation characteristics through controllable reconfigurable structures.

Based on different geometry and material combination, the soft metamaterials can be classified into two basic categories: (1) single-phase porous or cellular soft metamaterials^7,8^, and (2) multi-phase soft metamaterial with architectured reinforcement phase embedded in a soft matrix phase, such as layers or inclusions^9–11^. Recently, both the instability and wave propagation properties of the former class have been extensively explored. While, for the latter class, due to the complexity in material combination, only a few cases with simple reinforcements such as initially straight layers^12^ and circular inclusions^13^ were studied. For example, 3D-printed layered composites with hyperelastic constituents were shown to have dramatic instability-induced pattern transformation^14^ when subjected to uni-axial compression and therefore produce elastic wave-stop band^15^.

Here, we specifically focus on the architectured multi-phase soft metamaterial, considering the case where of a reinforcing hexagonal network is embedded in a soft matrix. It is known that honeycomb structures constructed of single phase materials can generate elastic wave band gaps^16,17^. However, the addition of the soft matrix significantly enriches the behaviour and tunability of the materials. For example, additional instability patterns that are not admissible without matrix materials can be achieved. As a result, the wave propagation properties can be tuned in a different range.

This paper describes the instability-induced pattern transformation and the corresponding elastic wave dispersion relations in hexagonal network reinforced composites under equi-biaxial compressive strains. The paper is organized into the following sections: in Section 2, instability-induced pattern transformation is explored via theoretical analysis and numerical simulations; in Section 3, elastic wave propagation analysis is performed for two distinct patterns; Section 4 concludes the work with summary and discussion.

## Instability-induced pattern transformation

**Figure 1:**
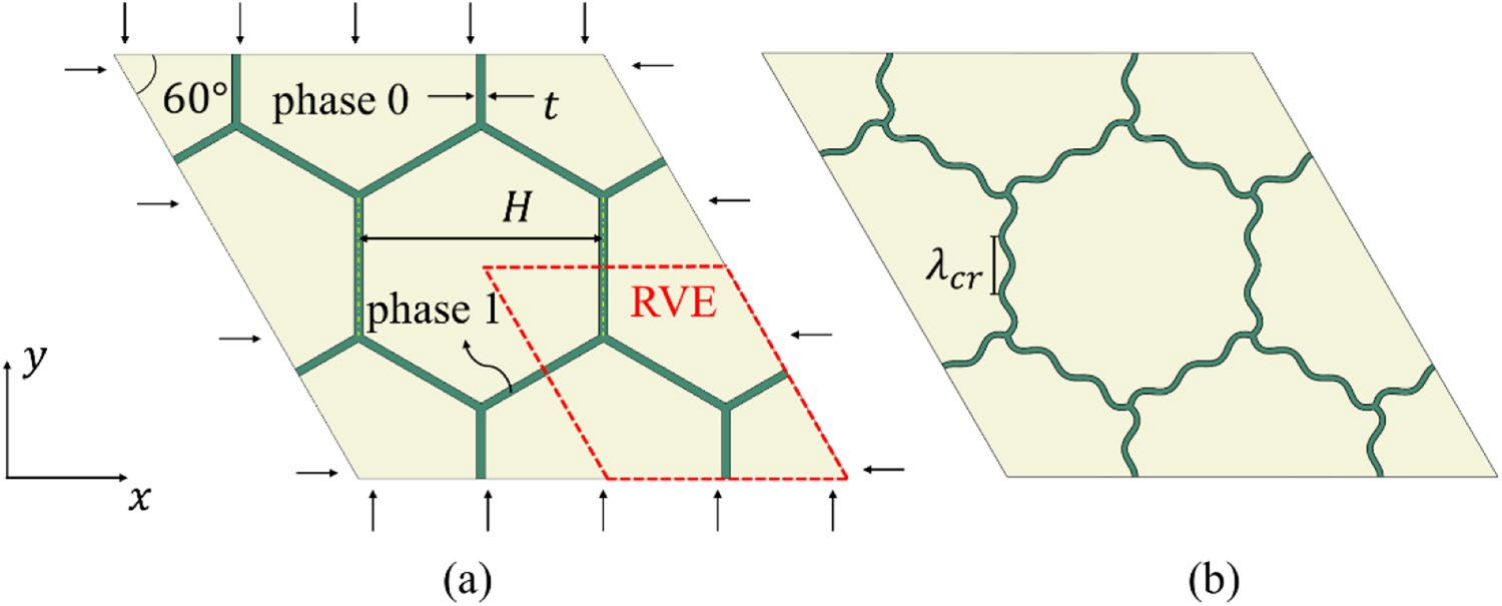
(**a**) The 2D RVE of the soft metamaterial with reinforcing hexagonal network embedded in soft matrix. The network (phase 1) has material properties (*μ*_1_, *v*_1_), while the matrix (phase 0) has material properties (*μ*_0_, *v*_0_). The thickness of the network walls is *t*, and the distance between the midlines of two opposite sides is *H*. (**b**) critical wavelength *λ*_*cr*_ of the cell wall under overall biaxial compression.

The geometry and material combination of the periodic composites is shown in Fig. 1. The 2D representative volume element (RVE) is framed in Fig. 1a. It can be seen that the periodic composites include two phases: phase 0, a soft matrix occupying the majority of the volume; and phase 1, a stiffer hexagonal network (phase 1) with thin cell walls embedded in the soft matrix. For phase 0, the initial shear modulus is ${\mu }_{0}$ and the Poisson’s ratio is ${\nu }_{0}$. For phase 1, the shear modulus is ${\mu }_{1}$ and the Poisson’s ratio is ${\nu }_{1}$. The thickness of network walls is $t$ and the size of hexagonal cell is determined by the distance $H$ between the midlines of two opposite sides. ${\lambda }_{{cr}}$ is the critical wavelength of the cell wall under the overall biaxial compression.

### Theoretical scaling law

When the applied equal-biaxial compressive strain reaches a critical value, the material microstructure can lose stability and the initial hexagonal pattern will be transformed to a different pattern. Governed by the balance between stretching energy of the matrix and bending energy of the hexagonal network, for the case of instability via ubiquitous wrinkling of the network walls, a scaling law for the critical compressive strain ${\varepsilon }_{{cr}}$ and the non-dimensionalized wavelength $\frac{{\lambda }_{{cr}}}{t}$ can be obtained by solving a forth order ordinary differential equation (ODE)^14,18^ as:

1 $${\varepsilon }_{cr}={{\rm{C}}}_{1}{(\frac{{\mu }_{1}}{{\mu }_{0}})}^{-\frac{2}{3}},$$

2 $$\frac{{\lambda }_{cr}}{t}={{\rm{C}}}_{2}{(\frac{{\mu }_{1}}{{\mu }_{0}})}^{\frac{1}{3}}.\,$$

where $${{\rm{C}}}_{1}=1.31{[\frac{(3-4{\nu }_{0})(1+{\nu }_{1})}{2{(1-{\nu }_{0})}^{2}(1+{\nu }_{0})}]}^{-\frac{2}{3}}$$, $${{\rm{C}}}_{2}=2.74{[\frac{(3-4{\nu }_{0})(1+{\nu }_{1})}{2{(1-{\nu }_{0})}^{2}(1+{\nu }_{0})}]}^{\frac{1}{3}}.$$

### Finite element simulations

Finite element models of the RVE were developed in ABAQUS V6.13. Equivalent bi-axial strains were applied, and periodic boundary conditions were applied on four edges of enlarged RVE. Both the network and matrix material were assumed to be neo-Hookean with shear modulus ${\mu }_{1}$ and ${\mu }_{0}$, respectively. For all FE models, ${\mu }_{0}=1MPa$; the densities $\rho$ of the constituents were chosen to be the same, $\rho$ = 10^3^ kg/m^3^, and the Poisson’s ratios were assumed to be 0.4 and 0.48 for the stiffer network and softer matrix, respectively. The networks were composed of regular hexagonal cells with a uniform size ($H=30\mu m)$, as shown in Fig. 1. FE simulations were performed by varying the thickness of the network *t* = 0.5, 1.2, and 4 *μm* and the stiffness ratio $\frac{{\mu }_{1}}{{\mu }_{0}}=50,\,100,\,1000.\,\,$2D plane strain elements (CPE8R) were used in the FE models To simulate both pre- and post- instability behaviors, the eigenvalue problem was first solved by using the ABAQUS/BUCKLE procedure, and then a small initial geometric imperfection (with the amplitude of 1% thickness of the interfacial layer) was introduced and the post-buckling analysis performed using ABAQUS/STANDARD.

**Figure 2:**
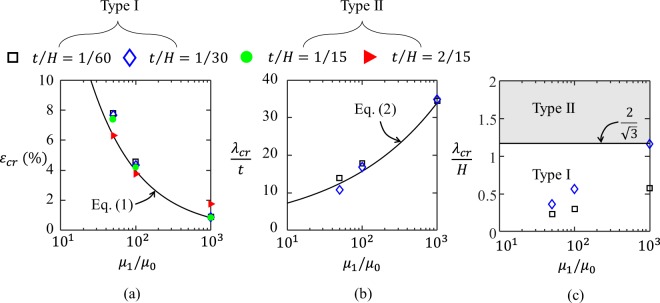
Comparison between analytical predictions and numerical results for different thickness to cell size ratios $\frac{{\boldsymbol{t}}}{{\boldsymbol{H}}}$ and shear modulus ratios $\frac{{{\boldsymbol{\mu }}}_{1}}{{{\boldsymbol{\mu }}}_{0}}$. (**a**) critical strain ${{\boldsymbol{\varepsilon }}}_{{\boldsymbol{cr}}}$ vs. $\frac{{{\boldsymbol{\mu }}}_{1}}{{{\boldsymbol{\mu }}}_{0}}$, and (**b**) nondimensional critical wavelength $\frac{{{\boldsymbol{\lambda }}}_{{\boldsymbol{cr}}}}{{\boldsymbol{t}}}$ vs. $\frac{{{\boldsymbol{\mu }}}_{1}}{{{\boldsymbol{\mu }}}_{0}}$. The hollow symbols represent Type I pattern. The solid symbols represent Type II pattern.

The theoretical and FE results of the equivalent critical strain ${\varepsilon }_{cr}$ and the non-dimensional wavelength ${\lambda }_{cr}/t$ are plotted as functions of wall thickness to cell size ratio $t/H$ and the shear modulus ratio ${\mu }_{1}/{\mu }_{2}$ in Fig. 2a,b, respectively. Due to the symmetric geometry and loading conditions of the model, the wave numbers of each segment of the network are the same for all cases. The numerical results are consistent with the theoretical prediction (Eqs (1,2)). Generally, according micro-instability and macro-instability, there are two types of instability patterns: Type I, micro-instability induced local wrinkling pattern (hollow symbols in Fig. 2a,b), and Type II, macro-instability induced global alternating pattern (solid symbols in Fig. 2a,b). To achieve Type I pattern, each edge of the hexagonal cell should be able to accommodate at least one-half wavelength ${\lambda }_{cr}$, i.e. $\frac{{\lambda }_{cr}}{2}\le \frac{H}{\sqrt{3}}$, as shown in Fig. 2c.

According to Eq. (2), the criterion in Eq. (3) must be satisfied for Type I pattern, otherwise, Type II pattern will be obtained.

3 $${(\frac{{\mu }_{1}}{{\mu }_{0}})}^{\frac{1}{3}}\frac{t}{H}\le \frac{1}{{{\rm{C}}}_{2}}.$$

**Figure 3:**
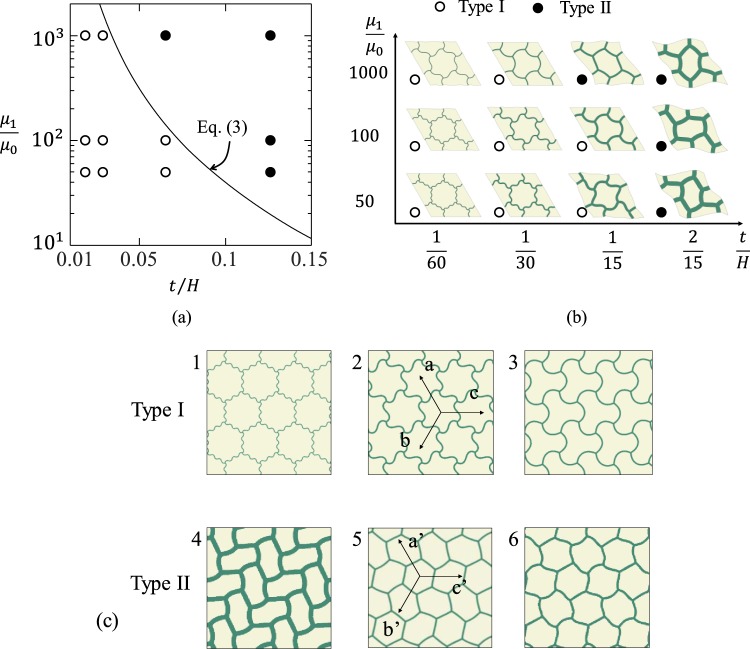
(**a**) Analytical model (black solid line) and FE results of the eigenmodes of hexagonal network reinforced composites as a function of thickness to cell size ratio ${t}/{H}$ and shear modulus ratio $\frac{{{\boldsymbol{\mu }}}_{1}}{{{\boldsymbol{\mu }}}_{0}}$; (**b**) FE results of the instability patterns of the hexagonal network reinforced composites. (Type I pattern is local repeating patterns (hollow symbols), and Type II pattern is global alternating patterns (solid symbols)); (**c**) summary of the two types of wrinkling patterns from numerical simulations. The details of material and geometric property of each case are follows: case 1($\frac{{\boldsymbol{t}}}{{\boldsymbol{H}}}=\frac{1}{60},\frac{{{\boldsymbol{\mu }}}_{1}}{{{\boldsymbol{\mu }}}_{0}}=50$), case 2($\frac{{\boldsymbol{t}}}{{\boldsymbol{H}}}=\frac{1}{30},\frac{{{\boldsymbol{\mu }}}_{1}}{{{\boldsymbol{\mu }}}_{0}}=100$), case 3($\frac{{\boldsymbol{t}}}{{\boldsymbol{H}}}=\frac{1}{15},\frac{{{\boldsymbol{\mu }}}_{1}}{{{\boldsymbol{\mu }}}_{0}}=100$), case 4($\frac{{\boldsymbol{t}}}{{\boldsymbol{H}}}=\frac{2}{15},\frac{{{\boldsymbol{\mu }}}_{1}}{{{\boldsymbol{\mu }}}_{0}}=50$), case 5($\frac{{\boldsymbol{t}}}{{\boldsymbol{H}}}=\frac{1}{15},\frac{{{\boldsymbol{\mu }}}_{1}}{{{\boldsymbol{\mu }}}_{0}}=1000$), case 6($\frac{{\boldsymbol{t}}}{{\boldsymbol{H}}}=\frac{1}{15},\frac{{{\boldsymbol{\mu }}}_{1}}{{{\boldsymbol{\mu }}}_{0}}=50$).

The FE results of the instability pattern for different thickness to cell size ratio $t/H$ and the shear modulus ratio ${\mu }_{1}/{\mu }_{0}$ are shown in Fig. 3a,b. When $\frac{{\mu }_{1}}{{\mu }_{0}}$ and $\frac{t}{H}$ increase, the instability pattern will transform from Type I to Type II. For the very large $\frac{t}{H}$ or $\frac{{\mu }_{1}}{{\mu }_{0}}$, the influences of the matrix become negligible, and the instability modes are asymptotic to the classical results of hexagonal honeycombs under equivalent biaxial compression^19^.

An exhaustive categorization of hexagonal network was proposed based on group-theoretic bifurcation theory^20^. The two types of patterns (shown in Fig. 3c) can also be defined according to the axes of symmetry and the number of cells in a smallest repeating unit of patterns. Type I pattern is the pattern of a single cell that repeats itself in all three axes of symmetry, thus the local pattern of one cell can represent the overall pattern. Type II pattern can be defined as one or several cell patterns alternating along one or multi axes of symmetry. The bottom row of Fig. 3c illustrates a couple of examples of Type II patterns. Image 4 has one cell pattern repeating along the b’ axis and two cell patterns alternating in the a’ and c’ axes; image 5 has two cell patterns alternating in all three axes, and pattern 6 has three cell patterns alternating in all three axes. In fact, as mentioned before, Type I pattern is due to micro-instability where only the cell wall wrinkles; while Type II pattern is due to macroscopic instability where the matrix and hexagonal network deform in a long-wave mode.

Type I patterns correspond to the critical half intrinsic wavelength equal to or less than the segment length; while Type II patterns correspond to the critical half intrinsic wavelength larger than the segment length (as shown in Fig. 3b).

**Figure 4:**
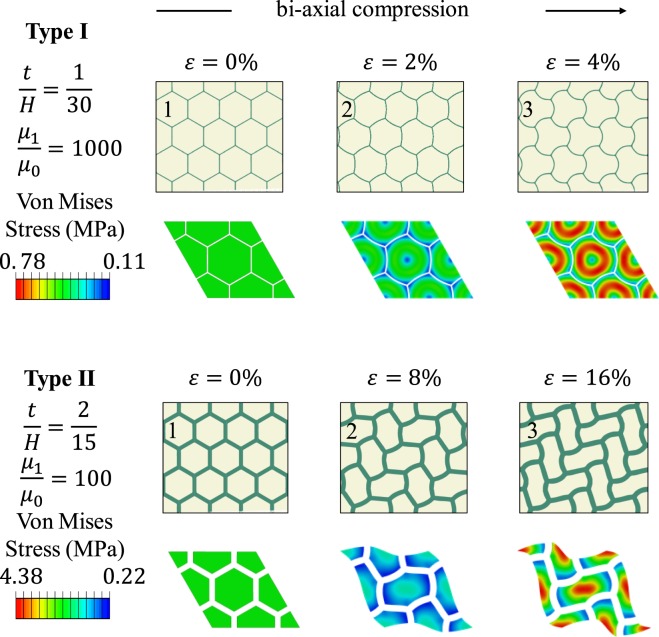
Evolution of wrinkling patterns for Type I ($\frac{{\boldsymbol{t}}}{{\boldsymbol{H}}}=\frac{1}{30},\frac{{{\boldsymbol{\mu }}}_{1}}{{{\boldsymbol{\mu }}}_{0}}=1000$) and Type II ($\frac{{\boldsymbol{t}}}{{\boldsymbol{H}}}=\frac{2}{15},\frac{{{\boldsymbol{\mu }}}_{1}}{{{\boldsymbol{\mu }}}_{0}}=100$) under three stages of equivalent biaxial compression (global strain *ε* = 0%, 2%, 4% (Type I), and *ε* = 0%, 8%, 16% (Type II)).

The evolution of Type I and Type II patterns are different under equivalent biaxial compression. As shown in Fig. 4, two representative cases were chosen: Type I ($\frac{t}{H}=\frac{1}{30},\frac{{\mu }_{1}}{{\mu }_{0}}=1000$) and Type II ($\frac{t}{H}=\frac{2}{15},\frac{{\mu }_{1}}{{\mu }_{0}}=100$). After the onset of instability, for Type I pattern, the wave amplitude increases, while for Type II pattern, the length aspect ratio of each cell increases. Interestingly, the contours of the Von Mises stress show that during the pattern evolution, for Type I pattern, matrix stress localized in the region close to the convex side of the wrinkled walls and the center of each cell has zero strain; while for the Type II pattern, matrix stress localized in the center of each cell. For both patterns, matrix stress is close to zero in the region close to the concave side of the wrinkled walls.

**Figure 5:**
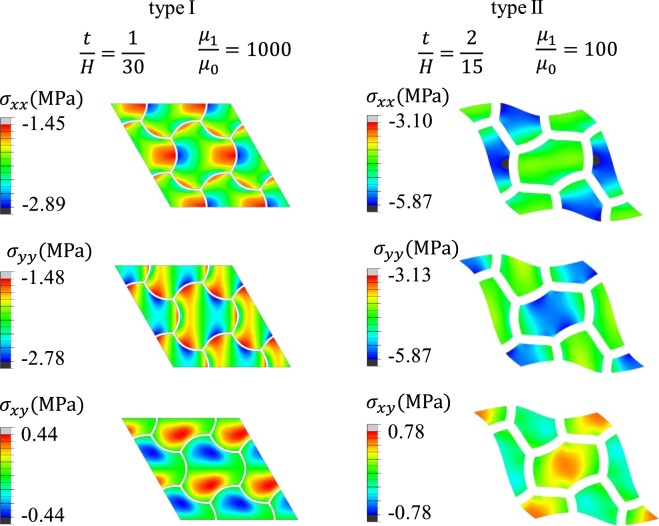
Normal and shear stress contours of type I ($\frac{{\boldsymbol{t}}}{{\boldsymbol{H}}}=\frac{1}{30},\frac{{{\boldsymbol{\mu }}}_{1}}{{{\boldsymbol{\mu }}}_{0}}=1000$) and II ($\frac{{\boldsymbol{t}}}{{\boldsymbol{H}}}=\frac{2}{15},\frac{{{\boldsymbol{\mu }}}_{1}}{{{\boldsymbol{\mu }}}_{0}}=100$) patterns.

The stress contours of the two representative Type I and Type II patterns are shown in Fig. 5 (with overall equal bi-axial strain $\varepsilon =4 \%$ (Type I) and $\varepsilon =8 \% \,$(Type II)). For the Type I pattern, the stress in the matrix reaches the maximum values close to the peaks and valleys of the wrinkled cell walls; while for the Type II pattern, the shear stress component reaches the maximum in the center of each cell.

Also, as shown in Fig. 5, for both types of patterns, the stress contours near the interfaces between the layers and the matrix shows that at those locations, the major stress components ${\sigma }_{xx}$ and ${\sigma }_{yy}$ are both negative, and the shear stress component ${\sigma }_{xy}$ is low. Therefore, for both patterns, debonding and/or large friction are not expected. This indicates another advantage for this material system.

**Figure 6:**
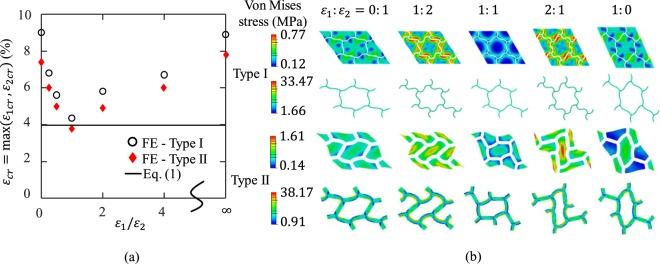
(**a**) The critical strain of instability for various bi-axial strain ratios, and (**b**) FE contours (with deformation amplification factor 2) of the matrix and the layers for Type I and Type II patterns, respectively.

To evaluate the influences of bi-axial strain ratio on the instability-induced pattern formation, two FE models were chosen for simulations under various bi-axial strain ratios. The two FE models are for two different cases under equi-biaxial loading, as shown in Fig. 3b: Type I pattern ($\frac{t}{H}=\frac{1}{30},\frac{{\mu }_{1}}{{\mu }_{0}}=100$), and Type II pattern ($\frac{t}{H}=\frac{2}{15},\frac{{\mu }_{1}}{{\mu }_{0}}=100$). For each model, simulations under seven bi-axial strain ratios were performed, i.e. ${\varepsilon }_{1}:{\varepsilon }_{2}=0:\,1,\,1:\,4,\,1:\,2,\,1:\,1,\,2:\,1,\,4:\,1,\,{\rm{and}}\,{\rm{1}}:\,0$. By defining the critical strain ${\varepsilon }_{cr}=\,{\rm{\max }}({\varepsilon }_{1cr},\,{\varepsilon }_{2cr})$, the influences of the bi-axial strain ratio on the onset of instability and the post-instability region for both cases are compared in Fig. 6a,b, respectively.

Fig. 6a shows that for both cases, the minimum critical strain for instability occurs at ${\varepsilon }_{1}:{\varepsilon }_{2}=1:\,1$, indicating that the equi-biaxial loading is the easiest loading case for the instability-induced pattern formation. When the bi-axial strain ratio increases/decreases beyond 1, the critical strain increases monotonically.

To explore the influences of the bi-axial strain on the two cases in the post-instability region, the FE contours of the maximum principal strain of the two cases at the same maximum overall strain (i.e. ${\rm{\max }}({\varepsilon }_{1cr},\,{\varepsilon }_{2cr})=10 \%$) are shown in Fig. 6b. In the soft matrix, for both types of patterns, the most-uniform deformation was generated under equi-biaxial loading. For cases with strain ratio other than 1:1, higher level strain concentrations were observed. Interestingly, for cases with ${\varepsilon }_{1}:{\varepsilon }_{2}=\,$1:2, and 2:1, the amplitude of the wavy layers for Type I pattern is larger than those of other cases; and for Type II pattern, the rotation angle of each cell is larger than those of other cases and the average strain in the layer are highest under equi-biaxial loading.

### Elastic wave dispersion relations and band gaps

**Figure 7:**
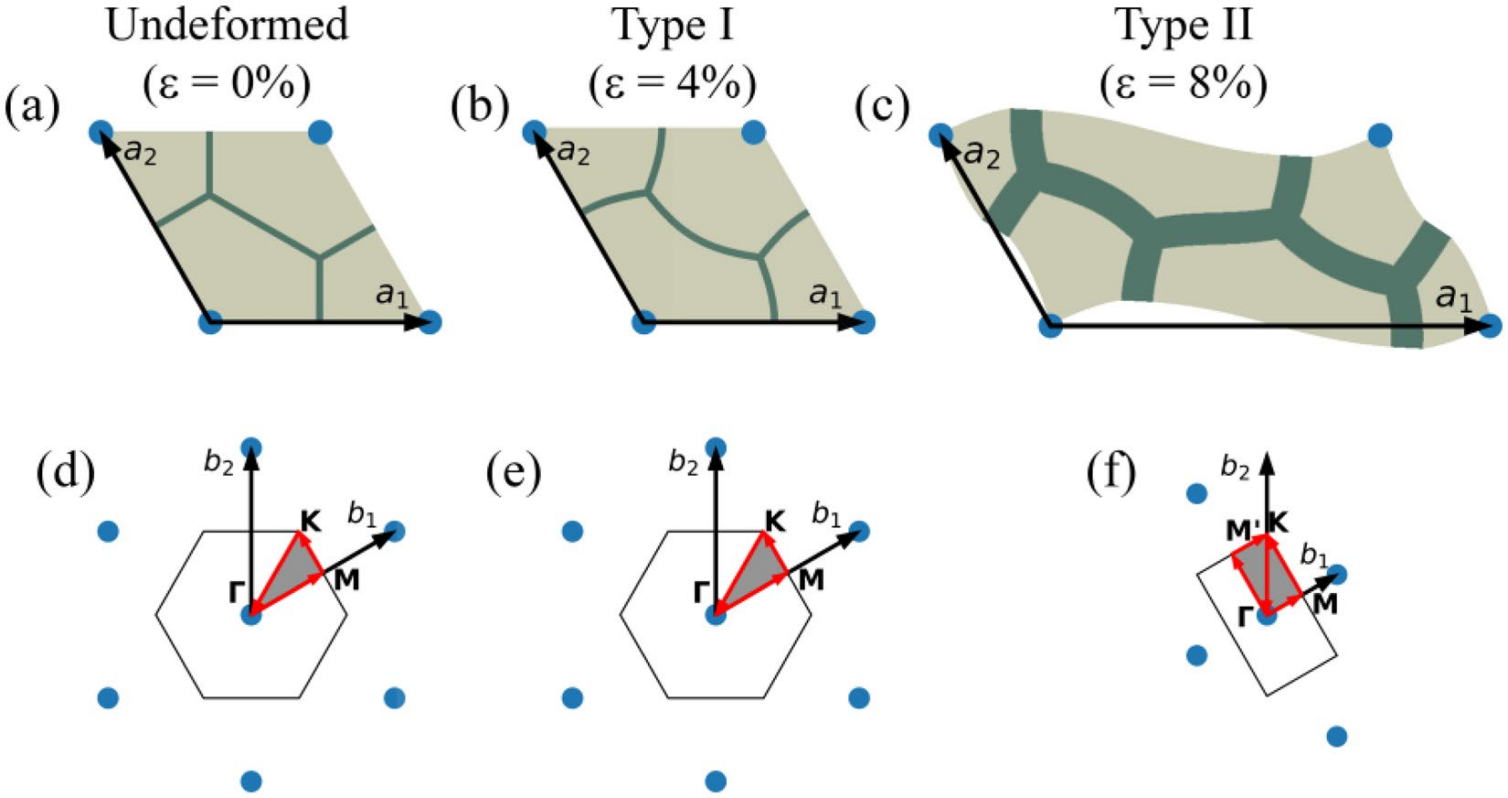
Primitive unit cells (**a,b,c**) and their reciprocal lattices (**d,e,f**) of the studied structures in undeformed and post-buckling state. Grey area represents the irreducible Brillouin zone (IBZ) and red arrows show the path along its perimeter.

Here we examine the acoustic properties of the soft transformable structures, and analyse the switchable behaviour induced by the instability induced pattern transformations. To study elastic wave propagation, the Bloch-Wave technique was utilized; in particular, Bloch-Floquet periodic boundary conditions were superimposed on the finitely deformed state of the composites^4,21,22^. First, the solution for finitely deformed structures in post-buckling regime was obtained; to this end, the initial geometric imperfections derived from the linear buckling analysis were introduced, and, then, equal bi-axial compressive strains were applied; so that the stress and strain states for each level of deformation can be obtained. Next, the Bloch-Floquet displacement conditions were imposed on the boundaries for different strain levels. The phononic band gaps in the undeformed state were identified by checking the eigenfrequencies for **k** vectors along the perimeter of the irreducible Brillouin zone (IBZ) of reciprocal lattice (see Fig. 7d–f).

Since the consideration is limited to the plane strain conditions, the dispersion characteristics of the longitudinal and in-plane transverse waves were analysed. Although the post-buckling composite structure remains periodic and the initially considered enlarged unit cell (Fig. 1a) still can be used to describe the formed patterns, this particular unit cell does not have the smallest area. While Bloch-Wave analysis can be performed on such unit cell, it usually leads to the appearance of reflected and artificial branches in the dispersion relations. To avoid this, a new representative unit cell is constructed; the new unit cell corresponds to the new “true” periodicity of the structure formed upon buckling. The constructed unit cell in the undeformed state (Fig. 7a) and after Type I buckling (Fig. 7b) coincide with RVE, framed in Fig. 1a; the new unit cell for Type II buckling is shown in Fig. 7c.

**Figure 8:**
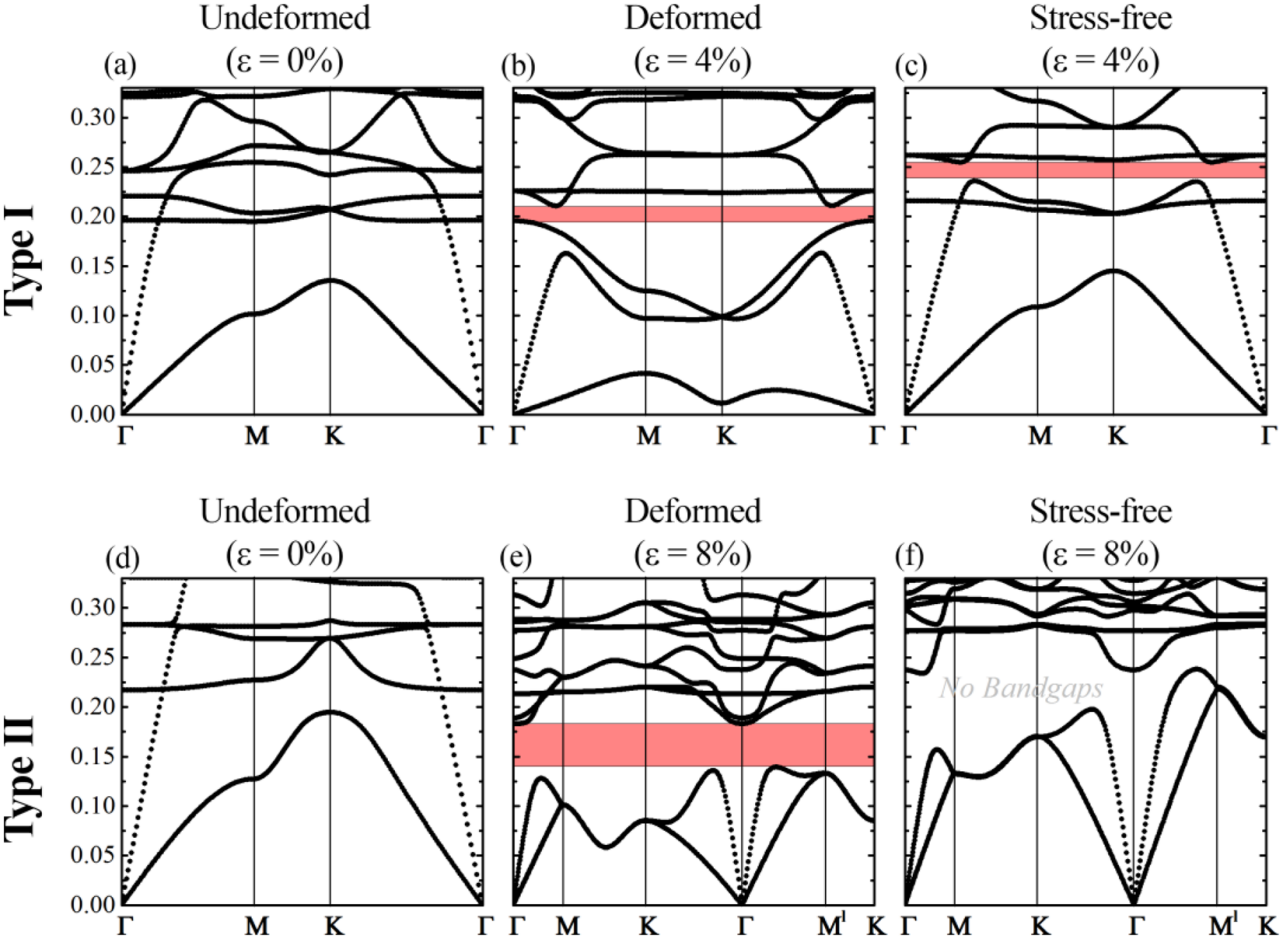
Band gap formation in the Type I (**a,b,c**) and Type II (**d,e,f**) patterns. The filled red area represents complete band gaps for in-plane waves. The Y-axis represents normalized frequency $f=\frac{\omega H}{2\pi }\sqrt{\frac{\rho }{{\mu }_{0}}}$.

Fig. 8a,b show the dispersion curves for the case of $\frac{t}{H}=\frac{1}{30}$ and $\frac{{\mu }_{1}}{{\mu }_{0}}=1000$ in the undeformed (a) and buckled states (b). For these geometrical and materials parameters, the soft metamaterial develops a local repeating pattern upon achieving the critical level of deformation. Since local Type I buckling retains the periodicity, the IBZs for the undeformed (Fig. 7d) and buckled states (Fig. 7e) coincide. As one may see from Fig. 8b, a local buckling within the periodic unit cell leads to the formation of complete (simultaneous shear and pressure) band gap. We should mention, however, that the width of the formed band gap is relatively narrow.

The instability induced formation of global alternating pattern (Type II) in the composite with $\frac{t}{H}=\frac{2}{15}$ and $\frac{{\mu }_{1}}{{\mu }_{0}}=100$ affects the elastic wave propagation differently. As one may notice comparing Fig. 7a,d and Fig. 7c,f, Type II buckling leads to the change in periodicity, and as a result, to the change of the basis vectors in the direct and reciprocal lattices, namely, vector ${b}_{1}$ decreases its length two times, while vector ${b}_{2}$ remains the same as the one in the initial configuration of the system. This leads to the change in the IBZ, which for the buckled case is highlighted in Fig. 7f. The formation of the global alternating pattern leads to the complete band gap, which opens in the range of relatively low frequencies. Remarkably, the width of this band gap significantly exceeds the width of the band gap that is formed due to local changes in the geometry of the unit cell (Type I). The same regularity was observed for cases with different geometrical and material parameters.

The applied bi-axial compression on par with accompanied buckling affect the composite state through geometrical changes and the formation of the internal stress state in soft matrix and stiff hexagonal network. In order to separate contributions of these two mechanisms, we performed Bloch-Wave analysis on the deformed *stress-free* primitive cells (Fig. 8c,f). Therefore, in these simulations only geometrical aspect of the buckling is taken into consideration. Comparing Fig. 8a and Fig. 8c we reveal that purely geometric rearrangement of the structure associated with Type I buckling does not have significant influence on the lowest branches of the dispersion curves due to relatively minor local alteration of the geometry. However, contribution of geometry is high enough to open a band gap as shown in Fig. 8c. This band gap is located at slightly higher frequencies as compared to the band gap observed after buckling (Fig. 8b); their widths, however, are almost identical. Therefore, for Type I buckling we observed that despite the significant change of the lower branches of the dispersion curves after buckling due to the internal stress state (compare Fig. 8b and Fig. 8c), formation of the band gap is associated mainly with the geometrical variation of the structure.

At the same time, opening of the band gap in the composite with Type II buckling is associated with a different mechanism. Comparing dispersion relation for post-buckling state (Fig. 8e) and stress-free deformed configuration (Fig. 8f), we reveal that while the internal stresses very weakly affects the shape of the lowest branches of the dispersion relation, their existence is essential for the opening of the complete band gap. Indeed, the band gap in the frequency range 0.15–0.18, which is observed in the post-buckling regime (Fig. 8e), does not exist in the stress-free case (Fig. 8f). Thus, the existence of internal stresses is a significant and essential factor enriching the tunability of acoustic properties and band gap formations in deformable composite metamaterials.

## Conclusions

In summary, finite element mechanical models of hexagonal network reinforced soft metamaterials were developed to systematically explore the influences of wall thickness and material combination on the instability of the material. Based on micro and macro instability, two types of patterns were formed: local wrinkling pattern (Type I) and global alternating pattern (Type II). Generally, when the wall thickness to cell size ratio $\frac{t}{H}$ increases and the shear modulus ratio $\frac{{\mu }_{1}}{{\mu }_{0}}$ decreases, Type I patterns can transform into Type II patterns. The criterion for pattern transformation is derived as Eq. (3). So that when $\frac{{\lambda }_{cr}}{H}\le \frac{2}{\sqrt{3}}$, Type I pattern occurs; otherwise, Type II pattern occurs.

Bloch wave analysis was performed for the two representative cases of Type I and Type II patterns. It was found that due to instability-induced pattern transformation, acoustic/elastic properties of the soft metamaterial can change significantly. In particular, complete band gap can be generated due to instability-induced pattern transformations. We revealed that variation of the elastic/acoustic properties is associated with two mechanisms, contribution of which depends on the buckling type.

By tailoring the geometry and material combination of the hexagonal network reinforced soft metamaterial, the band gap can be also tuned. For Type I buckling, even minor local changes in the geometry (under either stressed or stress-free conditions) may lead to opening of low frequency band gaps. While internal stress state significantly affects the lowest branches of the dispersion curves. For the representative case of Type II buckling, we showed that a combination of geometrical changes and existence of internal stresses is required to open wide band gap in the low frequency range.

We note that the development of macroscopic instabilities (Type II patterns) may be followed by a localized deformation, such as kink band; however, microscopic instabilities (Type I patterns) can develop through-out the material without failure under significant deformations. The distinguished behavior of very similar microstructures illustrates the rich properties that can be achieved through tailored design for switchable functionalities controllable by deformation.
